# Air Pollutant Particles, PM_2.5_, Exposure and Glaucoma in Patients with Diabetes: A National Population-Based Nested Case–Control Study

**DOI:** 10.3390/ijerph18189939

**Published:** 2021-09-21

**Authors:** Yun-Wei Chiang, Sheng-Wen Wu, Ci-Wen Luo, Shih-Pin Chen, Chun-Jung Chen, Wen-Ying Chen, Chia-Che Chang, Chuan-Mu Chen, Yu-Hsiang Kuan

**Affiliations:** 1 Department of Life Sciences, National Chung-Hsing University, Taichung 402204, Taiwan; apple3793@gmail.com; 2 Department of Optometry, Central Taiwan University of Science and Technology, Taichung 406053, Taiwan; 3 Department of Internal Medicine, Division of Nephrology, Chung Shan Medical University Hospital, Taichung 40201, Taiwan; shengwenwu1977@gmail.com; 4 The School of Medicine, Chung Shan Medical University, Taichung 40201, Taiwan; 5 Department of Pharmacology, School of Medicine, Chung Shan Medical University, Taichung 40201, Taiwan; kkjj88440@gmail.com; 6 Department of Pharmacy, Chung Shan Medical University Hospital, Taichung 40201, Taiwan; 7 Department of Internal Medicine, School of Medicine, Chung Shan Medical University, Taichung 40201, Taiwan; cshy752@csh.org.tw; 8 Department of Internal Medicine, Chung Shan Medical University Hospital, Taichung 40201, Taiwan; 9 Department of Education and Research, Taichung Veterans General Hospital, Taichung 40705, Taiwan; cjchen@vghtc.gov.tw; 10 Department of Veterinary Medicine, National Chung Hsing University, Taichung 402204, Taiwan; wychen@dragon.nchu.edu.tw; 11 Institute of Biomedical Sciences, National Chung Hsing University, Taichung 402204, Taiwan; chiachechang@gmail.com; 12 Department of Biotechnology, Asia University, Taichung 41354, Taiwan; 13 Department of Medical Research, China Medical University Hospital, Taichung 404333, Taiwan; 14 Traditional Herbal Medicine Research Center, Taipei Medical University Hospital, Taipei 110410, Taiwan; 15 Department of Life Sciences, National Chung Hsing University, Taichung 402204, Taiwan; chchen1@dragon.nchu.edu.tw

**Keywords:** PM_2.5_, glaucoma, diabetes, nested case–control study

## Abstract

The global prevalence of diabetes mellitus (DM) has reached 20%. Air pollutants with a particle size of less than 2.5 μm (PM_2.5_) are a globally recognized risk factor for diabetes and glaucoma. We examined whether the risk of glaucoma would decrease or increase when patients with DM were exposed to different PM_2.5_ concentrations. Data were obtained from the National Health Insurance Research Database (NHIRD) of Taiwan and the Air Quality Monitoring Network between 2008 and 2013. This nested case–control study involved 197 DM patients with glaucoma and 788 DM patients without glaucoma. Cases and controls were matched (1:4) by gender, age (±5 years), and index date (±6 months), and their data were entered in a logistic regression model adjusted for gender, age, urbanization level, income level, and comorbidities. The odds ratio (OR) of glaucoma at PM_2.5_ exposure concentration in the fourth quartile (Q4) compared with in the first quartile (Q1) was 1.7 (95% CI: 1.084–2.764). For glaucoma risk, the OR was 1.013 (95% CI: 1.006–1.020) at a PM2.5 exposure concentration in Q1, 1.004 (95% CI: 1.001–1.007) in the third quartile (Q3), and 1.003 (95% CI: 1.001–1.004) in Q4. In the subgroup analysis of patients living in non-emerging towns and non-agricultural towns, the OR for glaucoma in Q4 compared with in Q1 was 2.1 (95% CI: 1.229–3.406) and 1.8 (95% CI: 1.091–2.803), respectively (*p* *trend* = 0.001 and 0.011). For patients without migraine, the OR for glaucoma was 1.7 (95% CI: 1.074–2.782; *p* = 0.006). These results demonstrate that, for patients with DM, PM_2.5_ increased the risk of glaucoma and PM_2.5_ was an independent risk factor for glaucoma in patients with DM.

## 1. Introduction

In less than 30 years, the global prevalence of diabetes mellitus (DM) has reached 20% [1]. It is estimated that by 2040, approximately 642 million people worldwide will have DM [2]. PM_2.5_ (suspended particulate matter [PM] with a diameter of ≤2.5 μm) is a globally recognized risk factor for DM, and DM is a risk factor for glaucoma [3,4]. Experiments on mice have shown that PM_2.5_ induces the expression of hypoxia-inducible factor 1α, first causing retinopathy and then glaucoma [5,6,7].

Glaucoma is a neurodegenerative disease characterized by a slow degeneration of retinal ganglion cells over time [8]. The disease causes the inner layer of the optic cup of the eye to expand and the width of the edge of the neural retina to decrease [9]. The clinical features of glaucoma are diverse; some patients experience rapid onset or receive improper treatment, leading to short-term blindness, and others experience slow-onset, insidious glaucoma, which is difficult to detect [10]. Studies have demonstrated that PM_2.5_ is not associated with a risk of increased intraocular pressure (IOP), but others have revealed that higher air pollution levels increase the risk of glaucoma [11,12]. The complex relationship between PM_2.5_ and glaucoma is still unconfirmed, and no study has focused on the relationship between increased PM_2.5_ concentrations and an increased risk of glaucoma in patients with DM.

In 2016, there were 383 million people with diabetes worldwide, and about 1.4 million people died due to diabetes [2]. In addition, evidence has shown that type 2 diabetes (T2D) causes a series of chronic complications, including blindness, kidney disease and stroke, and then results in the huge burden on the healthcare system [13,14,15]. Long-term exposure to PM2.5 enhances the risk of the incidence of T2D by 11% in Taiwan [16]. Elderly patients are at increasing risk of PM2.5 exposure and primary open-angle glaucoma. More, the increase in the prevalence of primary open-angle glaucoma is based on exposure to higher levels of PM2.5 [17]. Air pollution is the main urban health risk factor causing approximately 7 million deaths each year worldwide [18]. The effectiveness of interventions in reducing ambient particulate matter air pollution reduces the impact of diabetes in America [19]. Therefore, we hypothesize that PM2.5 may indirectly increase the risk of glaucoma through its relationship with DM.

We examined whether the risk of glaucoma would decrease or increase when patients with DM had different PM_2.5_ exposure levels. This study analyzed the relationship between DM patients with and without glaucoma and their exposure to different concentrations of PM_2.5_.

## 2. Materials and Methods

### 2.1. Data Source

A secondary data analysis was conducted in this study. The National Health Insurance Research Database (NHIRD) holds the insurance data of 98% of Taiwan’s population of 23 million. The database was extrapolated under a program run by the National Institutes of Health. The Taiwan Longitudinal Health Insurance Research Database 2010 (LHIRD 2010) provides medical data on 1 million patients randomly selected from the NHIRD; this sample contains the health insurance information of patients from 2002 to 2013, including outpatient prescription drug treatment details (CD), outpatient prescription medical order details (OO), inpatient medical expenses (DD), covered data (ID), and basic medical institution details (HOSP).

PM_2.5_ Collection and Concentrations.

Data on PM_2.5_ exposure were provided by the Air Quality Monitoring Network (AQMN) established by the Environmental Protection Administration of Taiwan’s Executive Yuan [20]. Its directive was to monitor the air conditions in various regions of Taiwan, such as levels of PM_2.5_, PM_10_, carbon dioxide (CO_2_), Methane (CH_4_), Carbon monoxide (CO), Nitric Oxide (NO), ozone (O_3_), total hydrocarbon (THC), and other air pollutants [21]. A patient’s monthly average cumulative exposure from 2008 to 2013 of the observation periods was determined by the value recorded by the nearest station to their residence. The hourly cumulative exposure was multiplied by 24 h, and the average value was used as the basis for daily exposure. Daily exposure multiplied by the number of days in the month gives the average monthly exposure. If missing data for the observed daily exposure exceeded 8 h of data or if missing data for observed monthly exposure exceeded 10 days of data, these data were excluded. According to the AQMN data, we estimated the monthly average PM_2.5_ cumulative exposure concentrations. To confirm the exposure–response relationship, we consulted the results of a study in which researchers grouped PM_2.5_ exposure concentrations into quartiles and divided them into the following four groups: <25% (Q1), 0 to 561.56 μg/m^3^; ≥25% and <50% (Q2), 561.57 to 852.26 μg/m^3^; ≥50% and <75% (Q3), 852.27 to 1284.69 μg/m^3^; ≥75% (Q4), 1284.70 to 2727.44 μg/m^3^.

### 2.2. Study Population

**Figure 1:**
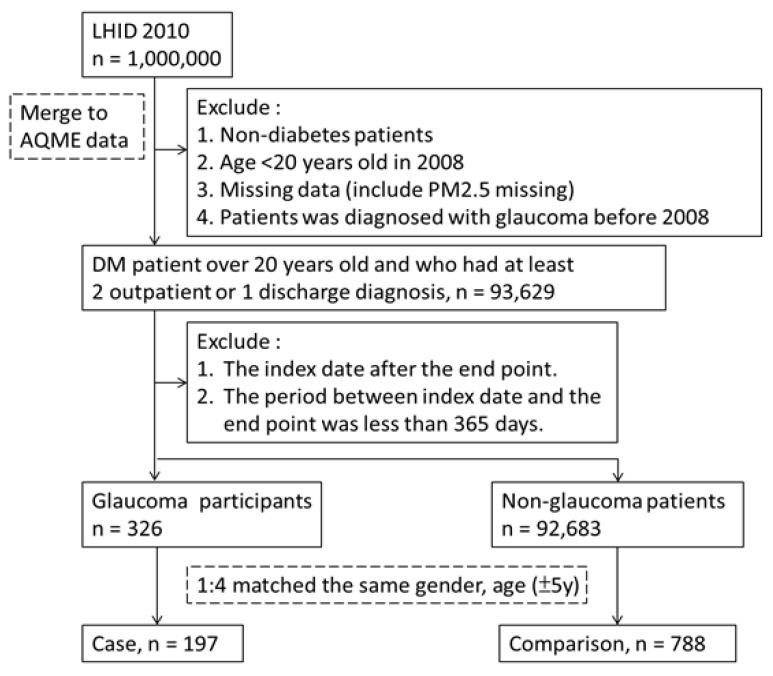
Flow chart of diabetes patients.

Participant data were sourced from the LHIRD and AQMN records between 2008 and 2013, and a nested case–control design was used. As shown in Figure 1, patients with DM were identified by the ICD-9-CM code 250.x, A181, having had three or more medical visits, or having one or more hospitalization records.

The index date refers to the date the patient was diagnosed as having DM. Patients with missing data, including gender, residential area, and PM_2.5_ values, were excluded. The included patients had DM concomitant with glaucoma (ICD-9-CM code 365.x) and three or more hospital visit records or at least a record of an outpatient department visit after the index date. We excluded patients who had glaucoma for less than 1 year (i.e., the period between the index date and the first diagnosis of glaucoma). The control group comprised patients with DM but without glaucoma. According to the nested research design, the control group was matched with matched individuals in the case group by gender, age (±5 years), and index date (±6 months) at a ratio of 1:4, and the initial glaucoma diagnosis date corresponded to the observational endpoint for the control. In total, the case and control groups respectively had 197 DM patients with glaucoma and 788 DM patients without glaucoma.

### 2.3. Comorbidities

Comorbidities of glaucoma as adjusted covariates include hypertension (ICD-9-CM code: 401.x–405.x), ischemic heart disease (410.x–414.x) and congestive heart failure (428.x), peripheral vascular disease (433.x), neurological disease, headache (784.x), migraine (346.x), epilepsy and recurrent (345), dementia (290), rheumatoid arthritis (714.x), asthma (Asthma, 493), chronic kidney disease (Chronic kidney disease, 585), fluid electrolyte acid-base disorders (276.x), hepatitis B (070.2, 070.3, V02.61), tuberculosis (010.x–017.x), anemia (280.x–285.x), peptic ulcer disease (531.x–534.x), depression (311), mental illness (298), and malignant disease (14.x–23.x) [22,23].

### 2.4. Statistical Analysis

The chi-squared test (χ^2^ test) was used to analyze the differences in the categorical variables between the case and control groups. We used the Shapiro–Wilk test to analyze data normality. The results were significant (*p* < 0.05), indicating the data was abnormally distributed. For abnormal distribution tests, the Wilcoxon rank sum test was used to check the differences in the categorical variables between the case and control groups. A multivariate logistic regression model was used to calculate the odds ratio (OR) and relevant adjustments, which include gender (female and male), low-income (incomes of less 20,000 New Taiwan dollars per month) [24], urbanization level (highly urbanized, moderate urbanization, emerging town, general town, aged township, agricultural town, and remote township) [25,26], and comorbidities, and then to assess the risk of glaucoma among patients with DM after PM_2.5_ exposure. A previous study indicated that the treatment demands of glaucoma are reduced by low-income situations [24]. The incidence of glaucoma is upregulated by urbanization level [25]. Finally, the multicollinearity was analyzed by a multiple linear regression test though the dummy variables including Variance Inflation Factor (VIF) and Tolerance Index (TI). A statistical analysis was performed using SAS 9.3 statistical software (SAS Institute, Cary, NC, USA), with *p* < 0.05 considered statistically significant.

## 3. Results

### 3.1. Demographic Characteristics

The data of 582 and 194 individuals in the control and case, respectively, were entered in this experimental analysis. Male patients accounted for 51.03% of the sample (Table 1).

No statistically significant difference was demonstrated in gender or average age between the case and control group (57.34 ± 10.39, 57.42 ± 10.56). In the case and control groups, 53.09% and 47.25% of patients, respectively, were from low-income households, but no significant difference was demonstrated. Those in the cases (33.51%) lived mostly in highly urbanized and moderately urbanized areas, whereas members of the control group (28.93%) lived in moderately urbanized areas (*p* < 0.05). Among comorbidities, most of the controls had higher rates of disease, but no statistically significant difference was found. No significant difference was demonstrated in the stratification of PM_2.5_ exposure concentrations. In the cases, the majority of people were in Q4, (31.47%), and in the controls, the majority were in Q2 and Q3 (25.13%).

### 3.2. PM_2.5_ Exposure Level and Glaucoma Risk

**Figure 2:**
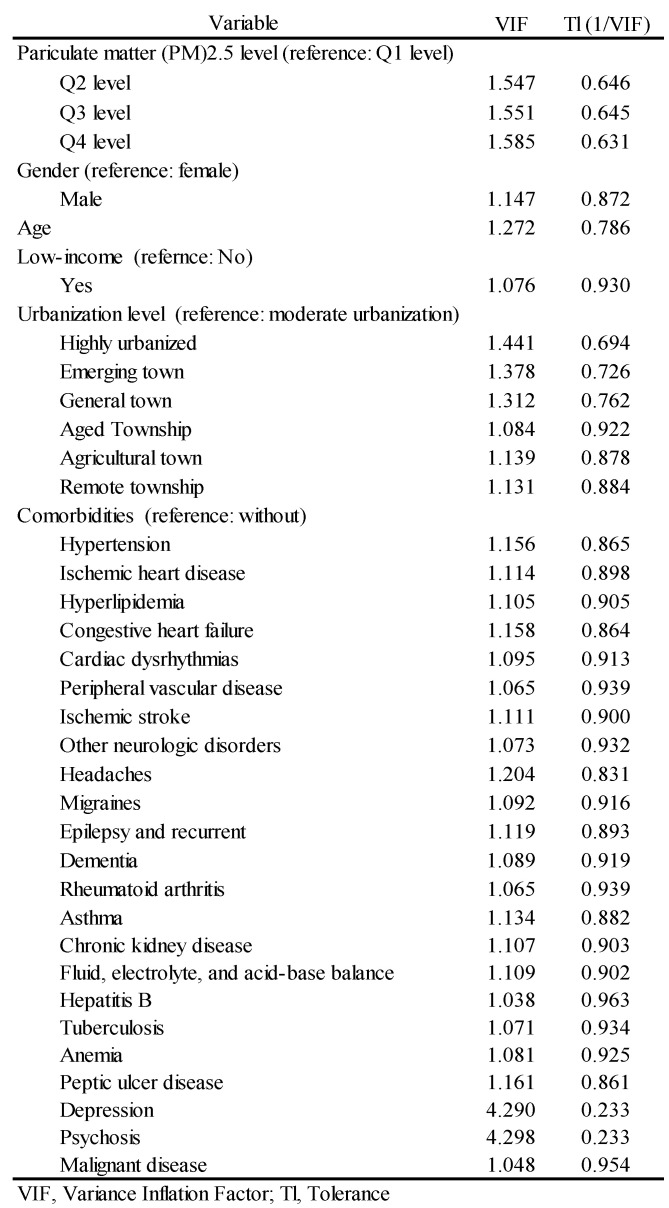
The standard (multiple) linear regression analysis model for collinearity diagnosis.

As shown in Table 2, after adjustments for gender, age, urbanization level, income level, and comorbidities, we found that the OR of PM_2.5_ exposure concentrations for glaucoma risk among participants in Q4 compared with Q1 was 1.731 (95% CI: 1.084–2.764), with no statistically significant difference between Q2 (OR, 1.232 [95% CI: 0.757–2.004]) and Q3 (OR, 1.451 [95% CI: 0.895–2.352]); however, a gradual upward trend was demonstrated. In terms of urbanization level, individuals living in emerging towns (OR, 0.559 [95% CI: 0.337–0.929]) and agricultural towns (OR, 0.197 [95% CI: 0.046–0.853]), when compared with those in moderately urbanized areas, had a lower risk of glaucoma and the difference was statistically significant. In terms of comorbidities, patients with migraine (OR, 2.672 [95% CI: 1.127–6.335]) had a higher risk of glaucoma, with statistically significant differences demonstrated. A VIF greater than 5 or a TI less than 0.20 predicted the occurrence of multicollinearity. Figure 2 shows that, in this research model, there is no collinearity among the variables.

**Figure 3:**
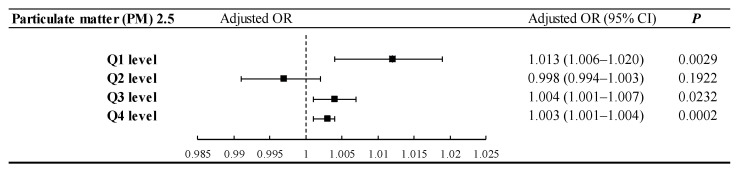
PM2.5 level subgroups analysis for the risk of glaucoma. Adjustment for gender, age, low-income, urbanization level, comorbidities. OR, odds ratio; CI, confidence interval.

As shown in Figure 3, after adjustment for gender, age, urbanization level, income level and comorbidities, statistically significant differences were noted; the OR for PM2.5 exposure concentration and glaucoma risk was 1.013 (95% CI: 1.006–1.020) for Q1, 1.004 (95% CI: 1.001–1.007) for Q3, and 1.003 (95% CI: 1.001–1.004) for Q4. Statistically significant differences were noted for individuals in Q2 (OR, 0.998 [95% CI: 0.994–1.003]). The ROC curve for predicting glaucoma had an AUC of 0.663 (95% CI: 0.618, 0.707) in diabetes patients.

Table 3 Subgroups logic regression analysis of Particulate matter (PM)2.5 level and Glaucoma. Adjustment for gender, age, low-income, urbanization level, comorbidities. OR, odds ratio; CI, confidence interval.

### 3.3. Independent Risk Factors

We conducted further subgroup analysis and stratification of statistically significant groups. In those living in the non-emerging towns, the risk of glaucoma at the Q4 exposure level was higher than at the Q1 level, and the difference was statistically significant (OR, 2.046 [95% CI: 1.229–3.406]; *p trend* = 0.001). The ROC curve for predicting glaucoma had an AUC of 0.666 (95% CI: 0.618, 0.715). In those living in nonagricultural towns, the risk of glaucoma at the Q4 exposure level was higher than that at Q1, and the difference was statistically significant (OR, 1.749 [95% CI: 1.091–2.803]; *p* = 0.011). The ROC curve for predicting glaucoma had an AUC of 0.630 (95% CI: 0.584, 0.676). In patients without migraine, the risk of glaucoma at Q4 was higher than at Q1, and the difference was statistically significant (OR, 1.728 [95% CI: 1.074–2.782]; *p* = 0.006). The ROC curve for predicting glaucoma had an AUC of 0.659 (95% CI: 0.613, 0.705).

## 4. Discussion

PM2.5 is a risk factor for glaucoma in DM patients. Epidemiological studies have become significantly important in this aging society with chronic disease. Economic growth and increased longevity have resulted in an increase in the older adult population. Elderly population growth has also increased the number of people with DM. Recently, the public has become increasingly aware of air pollution and its harmful effects [27].

Researchers have demonstrated that long-term exposure to PM_2.5_ can increase the risk of developing type 2 DM. As well as being linked to neurological diseases, PM_2.5_ is also strongly associated with DM [28,29]. Pregnant women, among other susceptible groups, are advised to reduce their exposure to PM_2.5_ for a reduction of DM risk [30]. Studies have indicated that long-term exposure to PM_2.5_ in Asian populations is strongly correlated with type 2 DM, and other studies have reported that people in areas with higher PM_2.5_ concentrations have a higher risk of developing DM than those in areas with lower PM_2.5_ concentrations [30,31]. Long-term exposure to PM2.5 leads to an increase in the incidence of T2D in North America and Europe, and it also confirms the increased risk of diabetes in Taiwan [16,17].

In the present study, we have found that individuals living in more urbanized areas have a higher risk of developing glaucoma. However, research in India has shown that the risk of developing closed glaucoma is higher in rural populations, while those patients with DM may have a higher risk of developing glaucoma. No statistical relationship has been established between air pollution and blood pressure disorders [32]. In urban areas, the open-angle glaucoma had a high prevalence due to the higher prevalence of myopia in these areas [33]. High myopia confers a high risk of developing glaucoma [34]. High myopia can arise due to numerous genetic and environmental factors and their interactions, leading to an increased risk of ocular complications including blindness and glaucoma [35,36,37]. One study found that the risk of myopia among 12-year-old schoolchildren in major cities in Australia was greater than that among children in suburban areas, and another study revealed that, among Vietnamese children, those in rural areas were more likely to have myopia than were those in urban areas [38,39]. Among adult men in Taiwan, advanced age, genetic factors (e.g., parental myopia), higher education, low outdoor activity level, and high urbanization led to a higher risk of myopia [40].

Although many factors can cause glaucoma, vascular changes play a key role in the pathophysiology of the disease [41]. Retinal vascular disorders and poor blood flow to the optic nerve head are associated with the manifestation of glaucoma [42]. In the blood vessels at the back of the eyeball, ocular blood flow in glaucoma patients is reduced and the resistance index is increased. Some researchers define migraine as a vascular and neurological disorder [43]. The pain associated with migraine is attributed to the activation of the trigeminal neurovascular system. The activation of nociceptors leads to the release of certain vasoactive peptides and inflammatory mediators, resulting in a reduction in cerebral blood vessel diameter. Changes in blood vessel diameter may relate to vascular disorders or vasospasms [44]. Therefore, the common underlying pathophysiological mechanism could be the link between glaucoma and migraine.

According to epidemiological studies, approximately 11% of individuals globally are severely affected by migraine, with a particularly high prevalence noted in the young population [45]. Here, the prevalence of age-related glaucoma was found to be similar to the prevalence of diabetes in the population. Regarding the correlation between PM_2.5_ and migraine, studies have revealed that exposure to higher concentrations of PM_2.5_ in the Taiwanese population increases the risk of migraine [46]. The literature is inconsistent on the association between glaucoma and migraine [47,48,49]. This study demonstrated that, among the population with diabetes, the degree of urbanization and migraine affect the risk of developing glaucoma. Studies have seldom explored the relationship between degrees of urbanization, migraine, PM_2.5_, and the risk of glaucoma. Our research revealed that exposure to higher concentrations of PM_2.5_ is an independent risk factor for glaucoma, suggesting a positive correlation between patients experiencing migraine and glaucoma. However, we maintain that DM is also a contributing factor in this complex interrelationship and is worthy of future discussion.

Studies have demonstrated that the retinal nerve fiber layer of patients with glaucoma is thinner than that of patients without glaucoma [50]. Central retinal artery occlusion, which often causes an increase in the thickness of the retinal nerve fiber layer, was reduced, and visual field defects were found in the corresponding position; they appeared to be closely related to the degree of structural damage [51]. In the present study, we proposed a positive correlation between PM_2.5_ and the development of central retinal artery occlusion. However, studies have shown that PM_2.5_ has no correlation with the IOP of patients with glaucoma. A possible explanation for this is that PM_2.5_ induces the structural change and diseases of the eye and therefore indirectly affects the development of glaucoma [11]. As shown in Figure 3, the predicted PM_2.5_ exposure level in Europe in 2006–2010 falls in Q2 [11]. No correlation between PM2.5 exposure at Q2 and glaucoma was uncovered in this study; more research is required. In addition, the results of our study show PM_2.5_ exposure levels divided into four quartiles. With an increases in PM_2.5_ concentration, the proportion of people with glaucoma is also increasing. Therefore, we conclude that PM_2.5_ is a risk factor for glaucoma in DM patients, and there is a concentration–dose relationship.

This study had some limitations as follows: (1) In terms of disease severity, we could not determine the exact blood sugar level in the population with diabetes, the severity of glaucoma, or the patients’ IOP; (2) Regarding patient environment, even with the nearest air pollution measurement station readings of PM_2.5_, it was not possible to determine each patient’s precise environment or to gauge the difference between outdoor and indoor PM_2.5_ concentrations; (3) Finally, patients’ living habits were not analyzed (e.g., exercise methods and eating habits); (4) We could not avoid “garbage codes” in our research. Glaucoma is not life threatening; while diabetes may present as a risk factor for cardiovascular or cancer deaths, it is less likely for either to be listed as the cause of death. So, this does not affect our results [52]. Nevertheless, the regulatory factors used in this article can explain similar results and are consistent with the results of multiple studies.

Our results indicated that PM_2.5_ exposure increases the risk of glaucoma in patients with DM. A higher cumulative exposure to PM_2.5_ results in a significantly higher risk of glaucoma. Therefore, the aging population and patients with diabetes-associated diseases should be aware of the harm caused by air pollution. By approaching methods such as wearing face masks and applying air purifying products in their daily lives, the chances of these diseases could perhaps be decreased.

## Figures and Tables

**Table 1:** Glaucoma and control from 2008 to 2013 in diabetes patients.

	Control		Glaucoma		*p*-Value
	(*n* = 788)		(*n* = 197)		
**Gender**					
Female	384	(48.73%)	96	(48.73%)	1.0000
Male	404	(51.27%)	101	(51.27%)	
**Age**					
Mean ± SD	57.35 ± 10.42		57.29 ± 10.34		0.9628
**Low income**					
Yes	366	(46.45%)	103	(52.28%)	0.1423
No	422	(53.55%)	94	(47.72%)	
**Urbanization level**					
Highly urbanized	210	(26.65%)	68	(34.52%)	0.0066
Moderate urbanization	228	(28.93%)	65	(32.99%)	
Emerging town	169	(21.45%)	27	(13.71%)	
General town	106	(13.45%)	22	(11.17%)	
Aged Township	10	(1.27%)	5	(2.54%)	
Agricultural town	40	(5.08%)	2	(1.02%)	
Remote township	25	(3.17%)	8	(4.06%)	
**Comorbidities**					
Hypertension	500	(63.45%)	136	(69.04%)	0.1428
Ischemic heart disease	83	(10.53%)	20	(10.15%)	0.5154
Hyperlipidemia	456	(57.87%)	124	(62.94%)	0.1953
Congestive heart failure	38	(4.82%)	4	(2.03%)	0.0828
Cardiac dysrhythmias	63	(7.99%)	20	(10.15%)	0.3296
Peripheral vascular disease	19	(2.41%)	5	(2.54%)	0.9177
Ischemic stroke	13	(1.65%)	2	(1.02%)	0.8759
Other neurologic disorders	11	(1.4%)	5	(2.54%)	0.2567
Headaches	266	(33.76%)	72	(36.55%)	0.4604
Migraines	19	(2.41%)	10	(5.08%)	0.0478
Epilepsy and recurrent	11	(1.4%)	1	(0.51%)	0.3094
Dementia	16	(2.03%)	2	(1.02%)	0.3413
Rheumatoid arthritis	21	(2.66%)	4	(2.03%)	0.6125
Asthma	166	(21.07%)	31	(15.74%)	0.0944
Chronic kidney disease	28	(3.55%)	7	(3.55%)	1.0000
Fluid, electrolyte, and acid-base balance	16	(2.03%)	3	(1.52%)	0.6431
Hepatitis B	43	(5.46%)	7	(3.55%)	0.2763
Tuberculosis	21	(2.66%)	2	(1.02%)	0.1702
Anemia	57	(7.23%)	21	(10.66%)	0.1112
Peptic ulcer disease	256	(32.49%)	61	(30.96%)	0.6824
Depression	12	(1.52%)	4	(2.03%)	0.6142
Psychosis	16	(2.03%)	5	(2.54%)	0.6591
Malignant disease	102	(12.94%)	26	(13.20%)	0.9245
**Particulate matter (PM) 2.5**					
Q1 level (0–561.56 μg/m_3_)	208	(26.40%)	39	(19.80%)	0.0686
Q2 level (561.57–852.26 μg/m_3_)	198	(25.13%)	46	(23.35%)	
Q3 level ( 852.27–1284.69 μg/m_3_)	198	(25.13%)	50	(25.38%)	
Q4 level (1284.70–2727.44 μg/m_3_)	184	(23.35%)	62	(31.47%)	

**Table 2:** Logic regression analysis of Particulate matter (PM)2.5 level and Glaucoma. Adjustment for gender, age, low-income, urbanization level, comorbidities. OR, odds ratio; CI, confidence interval.

	Glaucoma	
	Adjusted OR (95%CI)	*p*
**Particulate matter (PM)_2.5_ level (reference: Q1 level)**		
Q2 level	1.232 (0.757–2.004)	0.4015
Q3 level	1.451 (0.895–2.352)	0.1313
Q4 level	1.731 (1.084–2.764)	0.0215
**Gender (reference: female)**		
Male	0.835 (0.590–1.182)	0.3097
**Age**	0.999 (0.981–1.016)	0.8765
**Low-income (reference: No)**		
Yes	1.294 (0.927–1.806)	0.1299
**Urbanization level (reference: moderate urbanization)**		
Highly urbanized	1.109 (0.742–1.659)	0.6131
Emerging town	0.559 (0.337–0.929)	0.0247
General town	0.791 (0.453–1.381)	0.4104
Aged Township	1.795 (0.546–5.900)	0.3353
Agricultural town	0.197 (0.046–0.853)	0.0297
Remote township	1.138 (0.469–2.762)	0.7755
**Comorbidities (reference: without)**		
Hypertension	1.296 (0.898–1.872)	0.1665
Ischemic heart disease	0.930 (0.534–1.619)	0.7966
Hyperlipidemia	1.061 (0.750–1.500)	0.7372
Congestive heart failure	0.415 (0.137–1.258)	0.1200
Cardiac dysrhythmias	1.426 (0.803–2.531)	0.2255
Peripheral vascular disease	1.130 (0.387–3.306)	0.8228
Ischemic stroke	0.586 (0.120–2.855)	0.5086
Other neurologic disorders	1.930 (0.614–6.068)	0.2605
Headaches	1.232 (0.847–1.791)	0.2760
Migraines	2.672 (1.127–6.335)	0.0257
Epilepsy and recurrent	0.499 (0.058–4.271)	0.5258
Dementia	0.530 (0.112–2.513)	0.4238
Rheumatoid arthritis	0.770 (0.248–2.392)	0.6518
Asthma	0.665 (0.418–1.056)	0.0840
Chronic kidney disease	0.993 (0.397–2.487)	0.9884
Fluid, electrolyte, and acid-base balance	0.719 (0.187–2.770)	0.6321
Hepatitis B	0.716 (0.308–1.664)	0.4378
Tuberculosis	0.407 (0.090–1.839)	0.2425
Anemia	1.641 (0.933–2.887)	0.0855
Peptic ulcer disease	0.879 (0.605–1.279)	0.5015
Depression	2.403 (0.179–32.333)	0.5086
Psychosis	0.629 (0.062–6.395)	0.6955
Malignant disease	0.971 (0.597–1.580)	0.9071

**Table 3:** Subgroups logic regression analysis of Particulate matter (PM)2.5 level and Glaucoma.

		Particulate Matter (PM)2.5 Level (Reference: Q1 Level)			*p*
		Q2 level	Q3 level	Q4 level	
**Urbanization level**					
Emerging town groups					
Yes	(*n* = 196)	1.289 (0.320–5.190)	0.729 (0.168–3.166)	0.439 (0.080–2.423)	0.2572
No	(*n* = 789)	1.198 (0.695–2.066)	1.491 (0.871–2.550)	2.046 (1.229–3.406)	0.0018
Agricultural town groups					
Yes	(*n* = 42)	-	-	-	0.9729
No	(*n* = 943)	1.254 (0.768–2.049)	1.440 (0.883–2.349)	1.749 (1.091–2.803)	0.0117
**Comorbidities**					
Migraines groups					
Yes	(*n* = 29)	-	-	-	0.6125
No	(*n* = 956)	1.195 (0.721–1.979)	1.465 (0.893–2.403)	1.728 (1.074–2.782)	0.0065

## Data Availability

The data are not publicly available due to privacy or ethical restrictions.
